# Reviewed article: Guerreiro BA, Lara LS, Gialain IO, Silva CSV, Volpato LER. Analysis of the profile of patients admitted to an orofacial cleft rehabilitation service: an observational study, Cuiabá, 2005-2023. Epidemiol Serv Saude. 2025:34;e20240926

**DOI:** 10.1590/S2237-96222025v34e20240926.a

**Published:** 2025-10-24

**Authors:** Mariana Martire Mori

**Affiliations:** 1 Universidade Estadual de Maringá, Maringá, PR, Brazil

## Peer review A

## Questionnaire

Does the manuscript contain new and significant information to justify publication? No

Does the Abstract (Summary) clearly and accurately describe the content of the article? Yes

Is the problem significant and concisely stated? No

Are the methods described comprehensively? Yes

Are the interpretations and conclusions justified by the results? Yes

Is adequate reference made to other work in the field? Yes

## Manuscript Structure

Length of article is: Adequate

Number of tables is: Adequate

Number of figures is: Adequate

Please state any conflict(s) of interest that you have in relation to the review of this paper. None

Interest: Average

Quality: Below Average

Originality: Below Average

Overall: Average

Recommendation: Reject

## Comments

A introdução deve ser reescrita, contextualizando melhor o problema de pesquisa, e a caracterização mundial e nacional das fissuras labiopalatinas. O parágrafo que explica o local em que o estudo foi desenvolvido pode ser retirado da introdução e acrescentado ao método, como forma de caracterizar o cenário em que a pesquisa foi desenvolvida.

O objetivo da pesquisa precisa ser revisto. Considerem alterar o verbo “avaliar” para analisar.

No método não ficou claro a forma em que a amostra foi calculada e selecionada, e se houve necessidade de exclusão de algumas fichas (e os motivos que levaram à exclusão).

Descreva no método quais critérios foram utilizados para separar a análise em seis períodos.

Há necessidade de aprimorar a discussão e trazer os resultados encontrados na pesquisa em contraste com a literatura nacional e/ou internacional que encontraram informações semelhantes ou divergentes.

As conclusões devem ser revisadas para garantir que sejam justificadas pelos resultados e respondam diretamente ao objetivo, sem necessariamente repetir os resultados encontrados.

